# Investigation into psychological contract in ethically disciplined group: a case study of academics in Chinese higher education

**DOI:** 10.3389/fpsyg.2023.1157532

**Published:** 2023-07-20

**Authors:** Yao Fu, Yuan Xu

**Affiliations:** ^1^School of Foreign Languages Education, Shandong University of Finance and Economics, Jinan, China; ^2^School of Chemical Engineering, The University of Queensland, Brisbane, QLD, Australia

**Keywords:** psychological contract, ethical framework, job performance, ethically disciplined occupational group, professional ethics, organizational psychology

## Abstract

Ethical values and beliefs are increasingly realized as important factors in the operation of psychological contract for their potential role in determining individuals' attitudes toward employment relationships by valuing mutual exchange. However, to incorporate ethical terms into psychological contract analysis is challenging because they are often confused with relational contract, and ethics of professions can be difficult to summarize and interpret. This study has demonstrated how psychological contract operates within academics in Chinese higher education, an occupational group that is typically considered ethically disciplined and culturally bonded to their identity. Here, we designed a questionnaire survey focusing on transactional/relational psychological contract, ethical framework, and job performance, and statistically analyzed the responses to this survey from 230 Chinese higher education academics. It finds that the sample population perceived psychology contact with a relatively low contribution from monetary terms, while a strong correlation was observed between ethics and relational terms. In addition, the influence of emotional and ethical terms on job performance was clearly differentiated in statistics. From analyzing through a mediation model, it suggested an intermediated role of ethics between psychological contract and job performance. Findings in this study have demonstrated that ethically disciplined groups exhibit unique features in both their perceptions of psychological contract and their correlation with ethics and job performance, which is anomalous in other occupations. This study provides a protocol demonstrating the role of ethical framework in the operation of psychological contract, particularly within occupational groups bonded strongly to their identity/profession and constrained by ethics imposed by the society.

## 1. Introduction

The concepts of psychological contract create a methodological framework to analyze individuals' behavior within an organizational environment (Rousseau, 1989). This has been used widely in predicting employees' job satisfaction, dedication, and the intention to leave as well as identifying potential improvements in the sustainable development of organizations (Sturges et al., 2005; Cuyper and Witte, 2006; Gerber et al., 2012; Baruch and Rousseau, 2019). “Exchange” and “mutual benefits/obligation” are cores to studies based on psychological contract with a key underlying assumption that individuals/organizations would seek an equilibrium between mutual expectations (duties and rewards), which thus creates psychological ties between two parties leading to a pseudo-stable yet dynamic relationship. A healthy individual–organization interaction, often recognized as a balanced psychological contract, is beneficial for pursuing both the overall organizational goals and personal development (Jong et al., 2015; Lee and Chen, 2021; Mansur et al., 2021). As the subjective perception of employment pact, the psychological contract can be subtle. Employees and employers may not realize the existence of such a mutual expectation psychologically (which nevertheless determines their behaviors) (Shore and Barksdale, 1998), but account for specific issues of job dissatisfaction to more tangible factors, such as payment and working hours. Therefore, the psychological contract analysis within an organization is always not straightforward and requires various statistical approaches to decouple the complex individual–organization relationships.

With increasing interests in the theory of psychological contract in the changing society, an argument is made that it has emphasized the role of individuals' perception and psychological condition in determining their behaviors while overlooking the collective influence of considering employees as an occupational group (Bless, 2000; Seeck and Parzefall, 2008). This leads to psychological contract analysis having been more successful in industries with high turnover rates, where employees tend to behave on an individual basis. The relatively high turnover rate in those industries also means that the psychological contract exists for a shorter period of time, which makes it relatively superficial to interpret.

However, when one considers occupations with long-lasting convention and probably strong attribution to their identity, such as doctors, lawyers, scientists, engineers, and teaching professionals, the “contract” could be notably more stable and constrained (Bunderson, 2001; O'Donohue et al., 2007). Additionally, in such cases, the psychological contract is not limited to individual–employer relationship, but always extends to substantial ethical bonds to the society, the culture, and the country (O'Donohue and Nelson, 2009). Therefore, the willingness to behave according to certain ethical disciplines is a prerequisite of these occupations. These implicit rules (ethical disciplines) are intrinsically applied to individuals within these organizations, which are unlikely judged by the concept of “exchange” between individual and employer. This thus makes the approach of a psychological contract difficult to sufficiently understand and accurately predict the organizational climate relating to occupations that involve significant interactions beyond simple employment. Several factors have been used to improve the concept of psychological contract to incorporate the “group” or “collective” perception, such as the introduction of organizational commitment and citizenship behavior, community behavior, as well as approaches from ethical values and ideology (Turnley, 2003; Hui et al., 2004). The ethical and ideological terms are, however, rather subtle to realize in psychological contract analysis. It is particularly challenging to interpret these terms without an appropriate and extensive understanding of the conventional, historical, social, and cultural aspects of the occupation. This leads to very limited literature and an incomplete understanding in the area of psychological contract and organizational/individual behaviors within occupational groups that are ethically driven or disciplined.

Here, we approach this problem by conducting statistical analysis based on psychological contract schema, meanwhile aiming to decouple the ethical contribution from conventional “contracts.” We consider that the sample population, teaching professionals in Chinese universities, is an ideal and representative model system (both culturally and occupationally) to study the behaviors and psychological conditions of ethically disciplined groups in organizations. The particularity of this population will be discussed in the later sections. This thus forms a typical case study type of research where the key is to clearly identify the sample population and scope of the study. This study is significant and novel because, through a rationale that combines social, cultural, and political-economic perspectives, we have identified Chinese higher education academics as a particular sample population with clearly defined characteristics and ethical constraints, and, according to which, we designed questionnaire survey tailored to such an occupational group to statistically uncover their perceived psychological contract terms. The underlying hypothesis is that an in-depth analysis of a particular sub-group at the micro-level should gain valuable insights into describing the operation of broader social and organizational systems. Such case-study types of methodology have been extensively demonstrated in literature in economical, psychological, and social studies (Wu et al., 2019; Pöysä et al., 2021; Aluko et al., 2022; Zhang, 2022).

## 2. Literature review

### 2.1. Psychological contract in professional context

The concept of psychological contract is built from adopting an individual's cognitive and perceptual experience to describe the individual–organization relationship (Coyle-Shapiro and Conway, 2005). The underlying philosophy is that individuals believe that an organization reciprocates their contribution to obligation (or vice versa), which forms an unwritten contract between two parties reflecting mutual obligations (Pearce and Rousseau, 1998). The perceived mutuality in the psychological contract is “potentially idiosyncratic and unique to each person who agrees with it” (Rousseau, 1995), which makes the psychological contract inherently focused on an individual's subjective feelings/psychological conditions (Bellou, 2007). This thus provides a lever from a psychological perspective to analyze the individual's behavior within an organization, which has been shown useful and necessary in interpreting contemporary employment relationships.

The best-known established framework in approaching psychological contract consists of two-fold interpretive categories, namely, transactional contract and relational contract (Macneil, 1985; Rousseau, 1989). Transactional contract concerns mostly on self-interests (of both individual and organization), which is often represented by monetary value, such as rewards for extra working outputs, while relational contract focuses on the non-material or socio-emotional exchanges and tends to represent mutual benefits. The socio-emotional exchange builds up on utmost trust, implicit emotional attachment, and organizational commitment. Terms of psychological contract are a mental model as to what exchange-related behavior should and should not be, which naturally influences workers' attitudes, behaviors, and job-related outcomes. The belief that the counterpart has fulfilled obligations tends to result in positive attitudes and behaviors, giving rise to a satisfactory job performance and intention to stay (Turnley, 2003), while the perception of breached obligations often results in negative outcomes including turnover and neglect of job duties (Zhao et al., 2007; Amoah et al., 2021; Aluko et al., 2022).

### 2.2. Ethical frameworks in psychological contract

The documented bidimensional schema (transactional and relational) interprets the psychological contract as a single reciprocating relationship of exchange between individuals and the organization. An argument is that, from empirical and observational evidence (Marks, 2001; Dutton et al., 2010; Rust et al., 2016; Baruch and Rousseau, 2019), the relationship operates not only within but also outside the regarded organization. Works of literature have demonstrated that transpersonal contribution, in addition to transactional and relational, has been shown significant in creating psychological contract (Cullinane and Dundon, 2006; O'Donohue and Nelson, 2009), which largely refers to individuals' cognitive perception about how the work/relationship fits the entire society.

On the one hand, when a psychological contract is established, it is possible (empirically very likely) that individuals would take non-material forms of social ideological terms into valuing the exchange of contributions (Thompson and Bunderson, 2003). These ideological terms arise from individuals' belief or desire for highly valued principles or causes (e.g., the professional code and ethics) and have been shown to fundamentally govern the formation and operation of psychological contract, particularly in professional employees. However, these ideological terms are not explicitly included in the conventional bidimensional psychological contract schema. In studies that employed the concept of the psychological contract, it may be challenging to incorporate ideological contributions because those terms are often confused with the socio-emotional contributions of relational contract (Thompson and Bunderson, 2003; O'Donohue and Nelson, 2009), thus unable to form a distinctive element. Works of literature have created a refined interpretive framework by extending the psychological contract to an ideological/transpersonal contract with its interests exchange focusing on the society, the occupational group, or some kinds of metaphysical values (Bunderson, 2001; Thompson and Bunderson, 2003; Cullinane and Dundon, 2006). In contrast, the transactional contract focuses on self-interests while the relational contract focuses on the mutual interests between individuals and the organization. This tripartite psychological contract has opened up possibilities to identify and understand the role of ethical values in the operation of organizations and, more importantly, broadened the applicability of psychological contract to include third parties or contributions.

On the other hand, as an ideological term, ethical framework influences psychological contract in that it defines the criteria of individuals' decision-making, rather than being an elementary category of psychological contract. The fundamental of psychological contract comes from that “individuals use mental models both cognitively and intuitively to make meaningful interpretations of the intent of others, events and actions commonly encountered within the organization” (Rousseau, 2001). These “mental models” (often known as “ethical frameworks”) include the following: (1) individuals' knowledge, value, and belief, which originates largely from one's previous experience, education, and personal life; (2) the collective beliefs and values shared by the community and validated by peers and colleagues, as well as the professional code and the “ethical climate” within the organization or the society (Bloor and Dawson, 1994; Rousseau, 1995). The interaction between one's ethical frameworks and the psychological contract is complex, but, in brief, upon constructing a psychological contract, an individual would reason out the value of exchange based on his/her ethical frameworks (Victor and Cullen, 1988; Bloor and Dawson, 1994; Thompson and Bunderson, 2003). This means that the ethical framework serves as a criterion of individuals' decision on persisting or breaching the psychological contract, which consequently determines the dynamics of the psychological contract. For example, employees would reflect the deviations to psychological contract against both personal and collective ethical frameworks to reason whether they are acceptable and behave accordingly. As such, it is necessary to consider personal ethics and the “ethical climate” when studying and interpreting the psychological contract, especially for occupational groups with strong adherence to professional codes and ethics.

### 2.3. Job performance

Job performance symbolizes the extent to which individual employees help the organization achieve its goals (Pradhan and Jena, 2016). In contemporary theories of administration, the measures of job performance are typically based on their commitment to what they do, how well they do it, and the timeliness in doing it (Murphy and Shiarella, 1997). Conceptually and empirically, the division of job performance into two dimensions, namely, task performance and contextual performance, is generally accepted (Borman and Motowidlo, 1993). Task performance is about the proficiency with which a task is accomplished, while contextual performance enhances the working efficiency at both individual and organizational levels. Furthermore, contextual performance can be divided into interpersonal facilitation and job dedication (Motowidlo and Van Scotter, 1994; Van Scotter and Motowidlo, 1996). Interpersonal facilitation shows the interpersonal tendency that promotes collaboration among employees. It encourages and creates a context in which task performance achieves to facilitate the realization of organizational goals. Job dedication is mainly embodied in the initiative to solve problems at work, such as following rules, working hard, and self-discipline.

Teacher performance is the behaviors of teachers at work that can promote the completion of educational and teaching objectives. Task performance here refers to the professional achievements, including but not limited to educational and teaching activities as well as teacher-student interaction and communication, while contextual performance refers to the requirements beyond teaching duties, including work dedication, cooperation, and behaviors consistent with teacher morality (Hendrawijaya et al., 2020).

## 3. Sample population

### 3.1. Characteristics of academics in Chinese higher education

This article studies patterns of the psychological contract in the sample population of Chinese higher education academics, mainly teachers/teaching professionals in universities. We consider it a population that ideally demonstrates the operation of psychological contract in an ethically constrained occupational group. As such, an investigation into this clearly defined group of professionals can provide opportunities to test various hypotheses regarding the role of ethical framework in psychological contract and how it influences the individuals' behaviors in the work. In particular, we anticipate a particular and unique pattern of the psychological contract working with higher education academics in China because of the peculiarity of this group:

(1) In addition to “academics,” “researchers” or “university lecturers,” higher education workers in China are more broadly recognized as “teachers” (You, 2014), an occupation that has a long-lasting historical and cultural bond and commitment to society. The traditional understanding of teaching as a special mission of moral worth heaps of praise on teachers' moral roles and obligations to care for and help students. In Chinese culture and history (mainly Confucian's philosophy), a teacher does not only deliver knowledge or skills but also is regarded as a lifetime mentor and an ultimate model of “correct” behaviors, which even often extends from his/her own students to the general public (Han et al., 2016). Therefore, teaching has always been regarded as a profession of social value and teachers, as an identity, have enjoyed a highly respectable reputation and credibility in Chinese society for tens of centuries.
(2) Furthermore, the public usually, to some extent, imposes an idolized figure on teachers consistent with this reputation. The moral norms and codes of conduct that teachers are expected to abide by in their educational activities are highly emphasized, including their matching sentiments and qualities of always being righteous/continuous, compassionate, selfless and devotional, and the like (Ye and Zhou, 2020). This socio-cultural atmosphere is effectively a strong collective ethical framework (ethical climate) forcing teachers in China, making them refrain from deviation from these disciplines. Meanwhile, the ethical frame results in their inherent pride, which becomes the inherent potential constructive power corresponding to job performance. An occupation with such constraints may be similar to priests but in a more secular way. This thus means that the willingness to abide by these ethical constraints and to behave in accordance with them is actually a kind of social “license” to teachers in Chinese culture.
(3) Practically, job security and welfare are superior for Chinese teachers in state-funded institutes, especially higher education professionals. This topic is a different discussion than those frequently talked in Western universities, such as salary, paid leave, and superannuation (Gottschalk and McEachern, 2010; Heinz, 2015; Gander, 2022). With very few exemptions, state-owned universities and colleges in China are largely funded directly by the government, and cuts in educational budgets have never been heard for decades. Despite the increasing demands of becoming a university teacher, personnel redundancy is rare, which ultimately guarantees a kind of close-to-permanent relationship between academics and universities (Ye et al., 2022). In addition, teachers have the same high welfare as public servants in such aspects as medical care, health insurance, pension, and housing subsidies. While such situations of teachers in China are partly due to the historical/cultural aspects depicted in (1), they are nowadays further supported by the Chinese political–economic structure (Cao, 2009). This particularly forms an employment environment that is different from studies based on Western universities where a large number of fixed contracts and casual employees are involved.

From both historical/cultural and practical/realistic aspects, we consider academics in Chinese higher education to be a unique occupational group that bonds strongly to employers (universities) and the society, meanwhile, it is particularly constrained by their ethical frameworks. This thus effectively provides a model system to study the psychological contract outside the transactional and relational terms (i.e., the bonds to society, culture, and occupational identity) as well as the behavior of the ethically disciplined group. Note that the ethical values imposed on Chinese teachers are to some extent enforced and that the adherence to such disciplines is more rigid, which is different from the “ethical-driven” or “ethical-valued” group of occupation in literature, which refers largely to voluntary choices.

### 3.2. Demographical definition and statistical overview of sample population

This study specifically studied the subjects from three state-owned universities in eastern China. The sample was confined to university teachers who work on a full-time basis and returned fully completed questionnaires. The sample size consists of 230 valid responses. Of the respondent samples, 51.30% were women and 82.17% were married. The mean age of the sample was 42.4 years old. A total of 37.83% of respondents had teaching experience of more than 10 years. The mean teaching years was 15.7; 86.52% of them had an educational background of master's and higher. The composition of the sample at the professional level was as follows: 41.74% assistant professor and higher; 58.26% lectures and lower. Table 1 shows the demographical distribution and professional background of the studied sample population.

**Table 1:** Information of the samples.

**Title**	**Attribute**	***N***	**Percentage**
Gender	Male	112	48.70
	Female	118	51.30
Marital status	Married	189	82.17
	Unmarried	41	17.83
Educational background	Bachelor's and lower	31	13.48
	Master's and higher	199	86.52
Work experience	Less than 10 years	143	62.17
	More than 10 years	87	37.83
Professional level	Assistant professor and higher	96	41.74
	Lecturer and lower	134	58.26

## 4. Methodology

### 4.1. Research design

This study explores to construct the relationship models, analyzing qualitatively and quantitatively the influential factors and paths among psychological contract, teachers' ethical contribution, and how these reflect on their performance at work, taking Chinese university academic staff as the research subjects. It adopts descriptive research that is based on the survey analytical approach and is conducted from a positivist perspective. Adopting existing studies on related topics (Bathmaker, 1999; Tookey, 2013; Sewpersad et al., 2019), we created a questionnaire that was tailored specifically to university teachers. It began with personal and professional information, specifically, age, gender, marital status, educational background, working experience, and professional level. The questionnaire consisted of four parts, namely, perceived obligations of the university and teachers, respectively, in psychological contract, teachers' emotional status and ethical framework relating to psychological contract, and the anticipated job performance. Response to each question needed to be scored by the respondents. Rensis Likert scale (Likert, 1932) was employed to judge the direction and intensity of the participants' attitude, with the scores ranging from 1 to 5, respectively, indicating “not at all,” “slightly,” “somewhat,” “moderately,” and “to a great extent.” First, the relative scaling of questions was determined from preliminary interviews and trial surveys, on which the initial questionnaire was designed. According to the tests of reliability, some original questions were modified, and one question was removed. The second round of reliability tests as well as validity and factor analyses indicated the revised questionnaire was suitable for the survey. Next, a formal survey of a questionnaire with 59 questions was conducted in three universities in Eastern China focusing on teachers working on full-time arrangements from March to June 2022. Finally, the information data were gathered and analyzed through the administration of a questionnaire, using the statistical software SPSS 22.0.

### 4.2. Questionnaire design

The psychological contract inventory comprised two parts: university's obligations and teachers' obligations. According to Macneil's two categories (Macneil, 1985) of transactional and relational contracts adopted in a recent study (Baruch and Rousseau, 2019), the mutual obligations between universities and their employees were summarized into four aspects, respectively (detailed in Table 2); 14 questions were designed to describe university's obligations, e.g., “The university provides me with broad space for personal development and promotion,” “I have satisfactory compensation and benefits equal to my personal effort,” “My university attach importance to reasonable suggestions put forward by teachers”; while, in the same way, 11 questions were designed to describe teachers' obligations, e.g., “I seriously complete the tasks of teaching and research,” “I actively contribute to the development of the university,” “I feel it my duty to cultivate students' good ideological and moral character.” The higher the score, the higher the perceived degree of psychological contract fulfillment.

**Table 2:** Statistics of variables.

	**Variable**		**Mean**	**SD**
University's obligations in psychological contract	Economic/*monetary*	*T*	3.79	0.24
	Environmental/*organizational structuring and facilitation*	*T*	3.58	0.46
	Humanistic/*socio-emotional*	*R*	3.47	0.37
	Developmental/*career opportunities*	*R*	2.98	0.41
Teachers' obligations in psychological contract	Professional duty*/educational and teaching activities*	*T*	4.03	0.35
	Non-teaching duty/*service to the university*	*T*	3.76	0.47
	Attributional/*generalized occupational commitment*	*R*	2.87	0.37
	Social/*adherence to the occupational identity*	*R*	3.23	0.34
Job performance	Professional achievement*/job proficiency*		3.77	0.41
	Interpersonal facilitation/*cooperative activities*		3.81	0.60
	Job dedication*/work initiative*		4.01	0.46
Ethical framework	Satisfaction/*tenacity and optimism*		3.88	0.42
	Work–life balance*/psychological wellbeing at work*		3.27	0.34
	Intrinsic motivation/*ethical and devotional drive*		4.02	0.28
	Work recognition/*student and public appreciation*		3.45	0.27
	Personal and collective ethics/*social citizenship behavior*		2.53	0.61
	Ideological/*credible commitment to valued cause*		3.46	0.50

Words in italic are descriptive terms that relate the conceptualized variables specifically to university academics. For psychological contract terms, “T” and “R” refer to transactional and relational contracts, respectively.

Questionnaire revealing ethical value and emotional contribution was designed according to theories in an ethical framework (Kitwood, 1984; Bloor and Dawson, 1994; Tangney et al., 2007), their application in psychological contract analysis (Thompson and Bunderson, 2003; O'Donohue and Nelson, 2009), as well as the psychological wellbeing and subjective wellbeing (Diener et al., 2009; Bartels et al., 2019). Emotional contributions and ethical values are often linked together because out-directed emotions lead people to act ethically or unethically and vice versa (Thompson and Hart, 2006; Tangney et al., 2007), which makes them difficult to be differentiated through the simple descriptive approach adopted in this study. Therefore, this study considers these as a collective dimension that concerns how individuals align their values to the work. The questionnaire concerning ethical framework consisted of six variables (detailed in Table 2); 23 questions were designed for this part including “my work is recognized by others (such as university, students, and the public),” “My job makes it difficult for me to enjoy life,” “I feel that I'm responsible for students and their future developments,” “I restricted my personal social life because of being a teaching academic,” “I feel that I need to demonstrate correct behaviors and talking, particularly in front of students,” “My work is very important to the future of the society.” The negative emotion items were reversely scored. The higher the score, the stronger the perception of professional ethics and wellbeing at work.

Job performance inventory was based on the two dimensions (task performance and contextual performance) derived from literature (Motowidlo and Van Scotter, 1994; Van Scotter and Motowidlo, 1996) and modified to tailor teachers in three variables (detailed in Table 2); 11 questions were designed for this part, such as “My work always achieves the desired goal,” “I will help and support colleagues in their work when needed,” “I take the initiative to solve the difficulties in work.” The higher the score on the questionnaire, the better the job performance of the surveyed teachers.

### 4.3. Data collection and data quality

The questionnaire was distributed randomly and anonymously either via email or paper form. A total of 305 questionnaires were distributed and 246 were recovered, among which 230 were valid with an effective recovery rate of 75.4%. This gives a sample size = 230 for statistical analysis. Table 2 summarizes the statistics of variables collected from this survey.

To test the comprehensiveness and accuracy of the measured data, the returned valid questionnaires were subjected to reliability and validity analyses. The reliability of the questionnaire was tested by the α reliability coefficient method and Cronbach's α values of all variables studied in this work were >0.8, indicating a satisfactory reliability of the questionnaire. Table 3 illustrates the results of the reliability analysis.

**Table 3:** Reliability analysis.

**Variable**	***N***	**Cronbach's α**
Economic	4	0.831
Environmental	3	0.814
Humanistic	2	0.863
Developmental	3	0.842
Professional duty	5	0.829
Non-teaching duty	2	0.876
Attributional	2	0.811
Social	3	0.837
Professional achievement	4	0.819
Interpersonal facilitation	3	0.824
Job dedication	5	0.836
Satisfaction	3	0.819
Work–life balance	3	0.876
Intrinsic motivation	4	0.864
Work recognition	5	0.859
Personal and collective ethics	3	0.849
Ideological	5	0.867

Validity analysis included KMO and Bartlett's sphericity test and factor analysis. As shown in Table 4, KMO values were higher than 0.7 and Sig. = 0.00. The results bespoke that the questionnaire was valid and suitable for factor analysis.

**Table 4:** KMO and Bartlett's sphericity test.

	**KMO**	**Bartlett's test of sphericity**	
		**Approx. Chi-Square**	**Sig**.
Psychological contract: university obligations	0.738	3,557.937	0.00
Psychological contract: teachers' obligations	0.763	4,023.588	0.00
Job performance	0.871	2,387.482	0.00
Ethical framework	0.814	4,658.358	0.00

Table 5 was obtained after extracting the common factors of the questions in the questionnaire. All variables exhibited a cumulative variance >80%; and factor loading passed the 0.7 threshold for “psychological contract: university obligations,” while factor loading for other categories passed the 0.4 threshold. Such a communality evidenced by factor analysis indicated that the selected dimensions for the four investigative sections were reasonable and representative.

**Table 5:** Factor analysis.

	**Variable**	**% of variance**	**Cumulative % of variance**
Psychological contract: university obligations *Factor loading > 0.7*	Economic	24.724	24.724
	Environmental	27.601	52.325
	Humanistic	16.791	69.116
	Developmental	18.538	87.654
Psychological contract: teachers' obligations *Factor loading > 0.6*	Professional duty	23.258	23.258
	Non-teaching duty	14.863	38.121
	Attributional	20.557	58.678
	Social	21.682	80.360
Job performance *Factor loading > 0.5*	Professional achievement	27.225	27.225
	Interpersonal facilitation	39.047	56.272
	Job dedication	30.384	86.656
Ethical framework *Factor loading > 0.6*	Satisfaction	16.338	16.338
	Work–life balance	17.015	33.353
	Intrinsic motivation	10.579	43.932
	Work recognition	12.046	55.978
	Personal and collective ethics	11.637	67.615
	Ideological	13.155	80.730

### 4.4. Research hypotheses and models

Literature has shown that the perception of a psychological contract operating within one's ethical framework inspires a sense of emotional wellbeing and enhances job performance. At the same time, the feeling of doing correct things according to the ethical framework makes individuals tend to decide to persist with the psychological contract, improving its stability, which thus promotes job performance. This kind of mediation model with a dedicated indirect influence has been used in many studies in social, economic, and psychological areas to reveal the complex interaction between variables (Suazo, 2009; Al-Malki et al., 2022; Zhang, 2022). Therefore, we believe that, in addition to the direct influence on the psychological contract, individuals' perception of the ethical framework (as well as resultant emotional factors including wellbeing) plays a certain mediating role. This is in accordance with the theory of ethical framework depicted in Section 2.2. In other words, the impact of psychological contract on job performance exerts in two ways, one is through the psychological contract itself and the other is through the role of ethical values. Thus, we propose the following four hypotheses:

H1: job performance is positively influenced by psychological contract;
H2: ethical framework is positively influenced by psychological contract;
H3: job performance is positively influenced by the ethical framework;
H4: ethical framework plays a mediating role between the other two.
where the ethical framework being more positive means that individuals perceive increasing alignment of their ethical framework with the terms in the psychological contract.

The corresponding models of the above hypotheses are as follows:

M1: the impact of psychological contract as independent variables on job performance;
M2: the impact of the psychological contract as independent variables on ethical framework;
M3: the impact of the ethical framework as independent variables on job performance.
M4: ethical framework as an intermediary variable between psychological contract and job performance.

**Figure 1:**
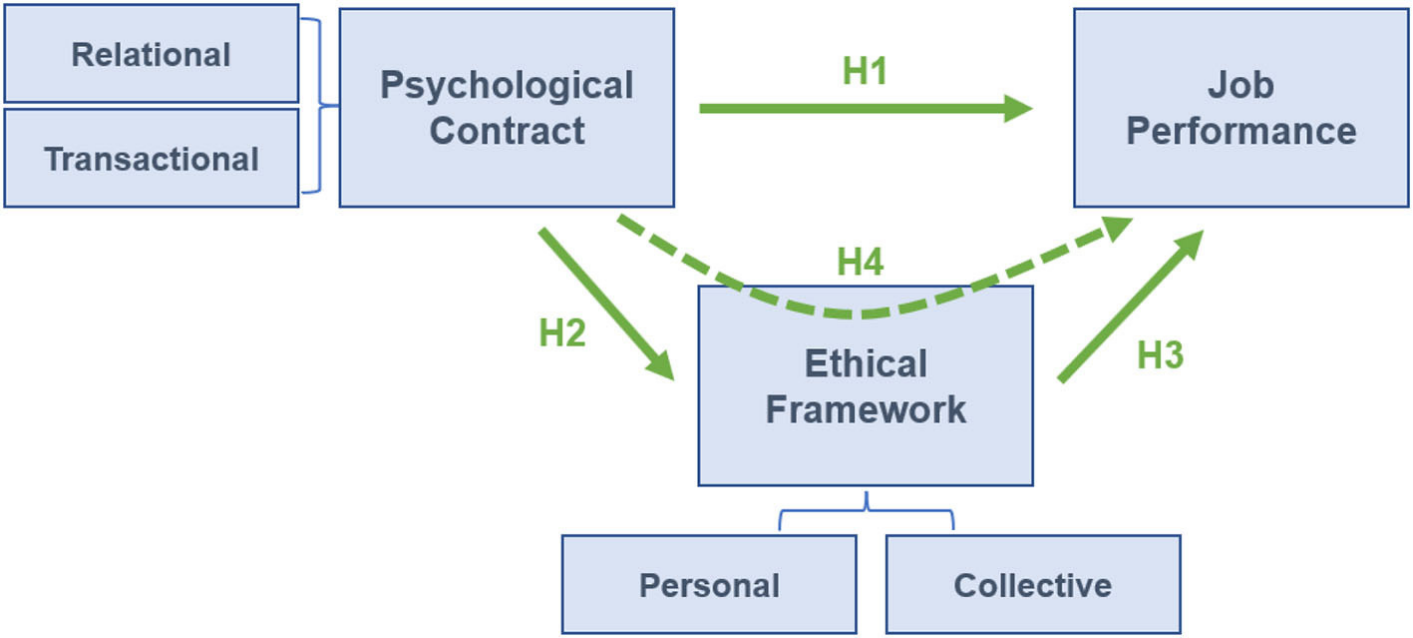
Schematics of investigated interacting model consisting of the psychological contract, ethical framework, and job performance.

As illustrated in Figure 1, the four models can further explore the overall interacting mechanism of investigated dimensions.

## 5. Statistical analysis and results

### 5.1. Correlation analysis

To explore the relationship and interaction between terms in the psychological contract, ethical framework, and job performance, a correlation analysis of all dimensions in this survey was conducted with results shown in Tables 6, 7.

**Table 6:** Correlation analysis of psychological contract on ethical framework and job performance.

**Variable**	**Economic**	**Environmental**	**Humanistic**	**Developmental**	**Professional duty**	**Non-teaching duty**	**Attributional**	**Social**
Professional achievement	0.493^***^	0.518^***^	0.379^**^	0.445^***^	0.555^**^	0.658^***^	0.563^***^	0.463^***^
Interpersonal facilitation	0.355^***^	0.325^***^	0.594^**^	0.617^***^	0.417^**^	0.489^***^	0.524^***^	0.638^***^
Job dedication	0.357^***^	0.484^***^	0.672^**^	0.667^***^	0.565^***^	0.354^***^	0.687^***^	0.619^***^
Satisfaction	0.349^**^	0.696^***^	0.391^**^	0.511^***^	0.497^**^	0.566^***^	0.435^***^	0.555^***^
Work–life balance	0.154^***^	0.554^***^	0.415^**^	0.397^***^	0.657^**^	0.711^***^	0.348^***^	0.347^***^
Intrinsic motivation	0.272^***^	0.358^***^	0.683^**^	0.617^***^	0.369^**^	0.387^***^	0.799^***^	0.421^***^
Work recognition	0.255^***^	0.497^***^	0.661^**^	0.698^***^	0.371^**^	0.384^***^	0.633^***^	0.62^***^
Personal and collective ethics	0.224^***^	0.587^***^	0.661^**^	0.745^***^	0.463^**^	0.548^***^	0.674^***^	0.636^***^
Ideologica**l**	0.235^***^	0.432^***^	0.871^**^	0.674^***^	0.327^**^	0.544^***^	0.688^***^	0.611^***^

^*^, ^**^, ^***^ represent data passed 90%, 90% and 95% confidence, respectively.

Data scale is colored blue (highest value) to yellow (lowest value).

**Table 7:** Correlation between ethical framework and job performance.

	**Professional achievement**	**Interpersonal facilitation**	**Job dedication**
Satisfaction	0.402^**^	0.354^***^	0.215^**^
Work–life balance	0.326^***^	0.241^**^	0.134^***^
Intrinsic motivation	0.529^**^	0.684^***^	0.54^**^
Work recognition	0.583^**^	0.497^***^	0.563^**^
Personal and collective ethics	0.528^**^	0.656^**^	0.526^**^
Ideological	0.568^***^	0.699^***^	0.587^***^

^*^, ^**^, ^***^ represent data passed 90%, 90% and 95% confidence, respectively.

Data scale is colored blue (highest value) to yellow (lowest value).

Noticeable findings are as follows:

- Among the interactions of the psychological contract, ethical framework, and job performance, the results presented that all correlations were positive.
- Economic aspect of university obligations offered a significantly low correlation with all variables in job performance and ethical framework (coefficients ~0.1–0.4). In the relationship of the three (Table 6), compared with a relational contract, the transactional contract of “professional duty” and “non-teaching” duty” of teachers as well as the “environmental” term of the university presented an overall weaker correlation with job performance and ethical terms (coefficients ~0.3–0.5 for both) in the yellow blocks.
- Furthermore, within the influence of transactional contract on the ethical framework, the relatively higher coefficients (0.696, 0.657, 0.711) correspond, respectively, to “satisfaction” and “work–life balance” of emotion, rather than to the rest of the terms of ethical values.
- In contrast to the above finding, ethical values (“intrinsic motivation,” “work recognition,” “personal and collective ethics,” and “ideological”) showed a significant positive correlation in the interaction with relational contracts for both university and teachers, which were social-emotional aspects. The vast majority of the coefficients here were between 0.6 and ~0.8, clearly illustrated by the blue blocks in Table 6.
- As is shown in Table 7, in the correlation analysis between ethical framework and job performance, emotional terms (“satisfaction” and “work–life balance”) displayed only a weak correlation with job performance (coefficients ~ general 0.1–0.3), with the weakest in job dedication. In contrast, ethical terms showed a much stronger correlation (coefficients in general >0.5), with the strongest in interpersonal facilitation. A boundary between emotional status and ethical values (blue-yellow denotes show high–low value) was clearly observed.

### 5.2. Regression analysis

The main purpose of this study was to establish whether a significant relationship exists between the independent variables and the dependent variables, which apply to this research. First, to test the collinearity, the variance inflation factor value (VIF) of each variable was calculated. Both maximum and mean values of VIF were less than the critical value 5, thus excluding the correlation anticipated between these variables that can threaten the validity of the regression analysis. Table 8 shows the result of the multicollinearity test.

**Table 8:** Multicollinearity test.

**Variable**	**VIF**
Economic	3.608
Environmental	3.055
Humanistic	2.533
Developmental	2.005
Professional duty	2.810
Non-teaching duty	3.665
Attributional	2.255
Social	3.548
Professional achievement	2.848
Interpersonal facilitation	3.155
Job dedication	4.515
Satisfaction	3.977
Work–life balance	3.477
Intrinsic motivation	3.04
Work recognition	4.049
Personal and collective ethics	4.099
Ideological	3.488
Mean VIF	3.290

Then, linear regression analysis was conducted for each dimension of job performance as a function of terms in the psychological contract. It was to analyze and numerically model the investigated variables to derive relationships between dependent and independent variables. Coefficients in the multivariable linear regression analysis model are presented in Table 9. The significant positive correlation between psychological contract and job performance validates our hypothesis 1, which forms the basis of this study.

**Table 9:** Regression analysis.

	**Professional achievement**	**Interpersonal facilitation**	**Job dedication**
Economic	0.336^***^	0.372^***^	0.292^***^
Environmental	0.293^***^	0.187^***^	0.267^***^
Humanistic	0.135^*^	0.241^**^	0.204^*^
Developmental	0.351^***^	0.297^***^	0.311^***^
Professional duty	0.339^**^	0.287^**^	0.298^**^
Non-teaching duty	0.164^***^	0.123^***^	0.097^***^
Attributional	0.216^**^	0.301^**^	0.342^**^
Social	0.226^**^	0.195^***^	0.138^**^
*R* ^2^	0.309	0.227	0.178
Adjusted *R*^2^	0.289	0.204	0.156

^*^, ^**^, ^***^ represent data passed 90%, 90% and 95% confidence, respectively.

### 5.3. Mediation model

In addition to the regression analysis (results in Table 9), which has tested a significant positive correlation between psychological contract and job performance, this paper further explores the possible mediated correlating scenario between psychological contract and job performance. As discussed in Section 2.2, an individual's ethical framework and resultant emotional status determine the decision-making and the dynamics of a perceived psychological contract. The ethical framework acts as a “judgment factor” during the forming, operating, and altering of the psychological contract, which eventually results in variations in job performance and outcome. This suggests a kind of mediated interaction between terms in the psychological contract and job performance, with the ethical framework being an intermediary variable. The validation of this hypothesis consists of testing the significance of regression coefficients in direct correlations (H1, 2, 3 in Figure 1) and mediated interaction (H4 in Figure 1), respectively. The simple regression equations are as follows, while the results are shown in Table 10:


(1) $$\begin{array}{l} jp=\alpha_{0}+\alpha_{1}pc \end{array}$$

(2) $$\begin{array}{l} ef=\beta_{0}+\beta_{1}pc \end{array}$$

(3) $$\begin{array}{l} jp=\gamma_{0}+\gamma_{1}ef \end{array}$$

(4) $$\begin{array}{l} jp=\delta_{0}+\delta_{1}pc+\delta_{2}ef \end{array}$$

**Table 10:** Mediation model.

**Coefficient**	**To job performance**	**To ethical framework**	**To job performance**	**To job performance**
	**(1)**	**(2)**	**(3)**	**(4)**
Ethical framework	–	–	0.479^***^	0.187^***^
Psychological contract	0.226^***^	0.398^***^	–	0.164^***^
*R* ^2^	0.058	0.067	0.070	0.073

^*^, ^**^, ^***^ represent data passed 90%, 95%, and 99% confidence, respectively.

*where pc, psychological contract; ef, ethical framework; jp, job performance. The “*α, β, γ, δ” *are coefficients of simple regression analysis*.

Figures in Table 10 show that:

(1) Respectively, the direct correlations between psychological contract, ethical framework, and job performance are positive, as well as that all the coefficients achieve >99% confidence.
(2) The result shows that the ethical framework influences job performance positively and that the framework is also positively dependent on psychological contract, indicating the potential mediation model. The magnitude and significance of the coefficient between ethical framework and job performance suggest a strong positive and constructive contribution from an individual's ethical values to job performance.
(3) The model of mediated influence (H4) incorporates the ethical framework as an intermediated variable between psychological contract and job performance. The multivariable regression (Eq.4) similarly results in positive coefficients with almost unchanged degree of confidence, while the absolute magnitudes of coefficients are reduced compared to the direct regression models. This validates the mediation model and suggests that the indirect effect (mediated via ethical framework) accounts for 0.329 (0.398 × 0.187 ÷ 0.226 = 0.329) of the influence of psychological contract on job performance.

## 6. Discussion

From the results presented in the previous section, this study creates a general view of how psychological contract operates within an ethically disciplined group exampled by the population of higher education academics in China. The statistical data show several characteristic features that potentially define this occupational group. We interpret the key findings from this study as follows:

- The survey design takes the positivist perspective approach; therefore, as expected, all correlations (shown in Tables 6, 7) between psychological contract, ethical framework, and job performance have been recorded as positive. This evidence that these correlations are constructive; i.e., positive terms in psychological contract promote the ethical framework and job performance, and vice versa. This is in general supported by literature.
- It is most noticeable (Table 6) that all entries in ethical framework and job performance exhibit significantly low dependency on the economic contribution in psychological contract, observed from the magnitude of correlation coefficients. This observation is anomalous compared to existing studies on psychological contract (including those conducted in university environments), where the reported discrepancies between dimensions are much narrower (Tookey, 2013; Aluko et al., 2022; Gander, 2022); i.e., psychological contract correlates to monetary terms similarly as other contributions. We consider this to be explainable according to the nature of this particular occupational group as depicted in Section 3. Higher educational academics in China are generally funded stably by the government, which means that fluctuations in an organizational environment (such as restructuring the management, redefining the job duty, and changing the assessment measures) or personal circumstances (such as emotions or workplace wellbeing) are unlikely to significantly influence the monetary income, as least not in an equivalent magnitude. Therefore, this causes the economic terms less significant in the psychological contract, which, at the same time, validates our descriptive figure/status about this occupational group. Extending from this argument, one can further interpret from the colored scale in Table 6 that, compared with relational contributions, the transactional terms are generally less correlated with job performance and ethical framework. This shows that the physical and tangible exchanges (transactional) within the psychological contract are not as influential as the socio-emotional and developmental (relational) counterpart. Such a discrepancy between these two categories of contract indicates the necessity to consider the ethical framework that fundamentally determines the perceived psychological contract, particularly in relational terms.
- Among terms in the ethical framework dimension (Table 2), “satisfaction” and “work–life balance” refer largely to optimism and general psychological wellbeing at work (i.e., how individuals consider/judge their psychological contract) (Wright and Cropanzano, 2000), while the rest terms refer to their ethical framework (i.e., how the psychological contract aligns with their ethical values) (Tov and Diener, 2009). It is not surprising to observe from Table 6 that “satisfaction” and “work–life balance” show the strongest correlation with the “environmental” term in the transactional contract of university obligations which includes organizational structuring, management, and facilitation to allow working efficiently and effectively. Also, it is interesting to find that these emotional terms, which are empirically considered relational (Turnley, 2003; Tangney et al., 2007; O'Donohue and Nelson, 2009; Rust et al., 2016; She et al., 2020), are more significantly correlating with a transactional contract in terms of teacher's obligations (professional duties and non-teaching duties). We anticipate that this correlation between personal emotion and psychological transactional contract suggests a kind of reciprocating constructive relationship; i.e., positive emotions promote the fulfillment of obligations, which in turn benefits the emotional status.
- Terms associated with ethical values of academics illustrate a significant correlation with relational psychological contracts regarding both universities and teachers' sides shown in Table 6. This agrees with previous studies (Barnett and Schubert, 2002; Turnley, 2003; O'Donohue and Nelson, 2009), as aligning one's ethical framework with an organization's ethical climate is one of the natures of the perception of a relational contract. The strong correlation recorded here possibly suggests a significant three-party interaction between psychological contract, ethical framework, and job performance, which means an inter-penetrating influence of psychological contract and ethical framework on job performance. Therefore, it is necessary to test our hypothetical mediated model through multivariable regression analysis.
- Theories in psychology indicate that emotional status and individuals' ethical values in work are often inter-connected (Barnett and Schubert, 2002; Tangney et al., 2007; Pöysä et al., 2021) and thus analyzed as a collective variable, because individuals tend to be more emotionally content when they think they are doing a correct thing. In this study, Table 7 particularly shows the relationship between emotion/ethics and job performance, where an interesting finding is that ethical terms exhibit distinct effects on job performance. Emotional terms (“satisfaction” and “work–life balance”) displayed only a weak correlation with job performance, especially in job dedication, whereas ethical terms show a much stronger correlation. It may be surprising to believe that the individual's self-content influences rather negligibly on the job performance, but we consider this exactly defines the behaviors of what we named “ethically disciplined” group. For this special occupational group, ethics and ideological values are largely imposed and regulated by the outer social and organizational environment (as discussed in Section 2.2). Teachers have adhered strongly to the identity or the “license to operate,” which consequently suppresses the expression of self-interests and emotions. In other words, statements like “I don't want to perform my teaching duty because I'm not happy with my work/working environment” will be regarded as unacceptable, and actually disgraceful, in the current social climate in China. As such, findings in Table 7 explain the reasons and necessity for studying this “ethically disciplined” group, which is fundamentally different from the “ethically driven” model, because the ethics are more restrictive and constrained to individuals' behaviors and thinking patterns.
- Given the strong correlation between ethical terms and job performance shown in Table 7, our mediation model (results summarized in Table 10) shows that a non-negligible proportion (>30%) of the influence of psychological contract on job performance arises from the ethical framework. This has deconvoluted the contributors from the psychological contract and ethical framework to an individual's performance at work and validates our hypothesis that the ethical framework is an intermediary factor between the psychological contract and job performance. In other words, it shows that ethical values take a significant role in psychological contract to achieve its functionality in an ethically disciplined population and vice versa. The result has been proved by the online courses and resultant restructuring of teaching activities that become a major problem of demotivation and demoralization in university teachers due to the lack of emotional exchange with students during the COVID-19 pandemic, during which the present study was conducted, and the data were collected. This situation could inevitably lead to alterations in psychological contract terms and teachers' daily behaviors at work (Garcia-Alvarez et al., 2021). However, the strong role of the ethical framework demonstrated in this triadic relation forms another characteristic of this occupational group whereas difficult to be revealed through conventional approach in psychological contract (transactional and relational). It makes sense to consider the ethical framework of intrinsic motivation as an integrated factor in exploring the interactions.

## 7. Conclusion and recommendations

This study has explored the impact of psychological contract on job performance and the mediating role of ethical framework in ethically disciplined occupational groups exampled by academics in Chinese higher education. Our results illustrate a few key features of the operation of psychological contract within such an occupational group and show how it interacts with an ethical framework. Specifically, these include the following:

(1) Dependency on monetary terms in psychological contract is relatively low.
(2) Correlation between relational contract and ethical framework is strong and significant.
(3) Job performance correlates strongly with ethical values, but weakly with emotional contributions.
(4) Ethical framework significantly mediates the influence of psychological contract on job performance.

We believe these characteristics stem from the cultural/social background of the teacher as an identity in China, as well as the political–economic status of this particular occupational group, which have made academics in Chinese universities dependent financially less on their employer (universities) whereas bonded strongly to ethical values imposed by the society. Additionally, the complexity of this group also lies in that its work integrates self-discipline and heteronomy, creation and regulation, individual and collective, and immediate and delayed effect. Occupational uniqueness determines that systems alone cannot meet various psychological needs leading to a sense of occupational wellbeing. In this case, the ethical framework qualitatively different from that of other occupations plays a vital mediating role. These particularities make Chinese higher education academics an ideal sample population to study the psychological contract within an ethically disciplined group. This study provides an improved understanding of how ethical values can be incorporated into concepts of the psychological contract, which possibly extends to occupational groups with similar adherence to their identity and bonds to the society, such as police, military, and medical professionals. We highlight the finding in Table 7 where a clear differentiation is observed between ethics and emotion (blue and yellow regions), which forms a characteristic of this occupational group and should be certainly pursued by further studies to better distinguish these contributions and interconnections. Future studies should also proceed toward investigating the ethical framework in more complex patterns of psychological contract models, such as those including organizational climates and collective behaviors.

This study lays the groundwork for studying the role of ethics within psychological contract. Professional ethics and adherence to the identity are critical for certain occupations, which must be considered when planning a restructuring of the working organization because they are effectively “contracted” stronger with society and profession than with their employers. This means that status of psychological contract within these occupations may react sensibly with the ethical values shared by the society (e.g., recognition and appreciation from the general public for their works and contributions to the better future of the society), instead of within an organization. In particular, we anticipate this will provide a useful reference to the educational policymakers that possibly inspire innovations in management, organization, and development of teaching and academic occupations. As such, fluctuations in psychological contract frameworks responding to the social atmosphere should be aware to avoid disruption in academics' individual careers, as well as the sustainable development of educational organizations.

## Data availability statement

The raw data supporting the conclusions of this article will be made available by the authors, without undue reservation.

## Ethics statement

Ethical review and approval was not required for the study on human participants in accordance with the local legislation and institutional requirements. Written informed consent from the (patients/participants OR patients/participants legal guardian/next of kin) was not required to participate in this study in accordance with the national legislation and the institutional requirements.

## Author contributions

YF formulated the idea, designed the study, collected data, and drafted the initial writing of the manuscript. YX analyzed the data and revised the initial writing of the manuscript. YF and YX worked together in further data analysis, final writing, and constant revision. All authors contributed to the manuscript and approved the final version submitted.

## References

[B1] Al-Malki A. M. Hassan M.-U. Ul-Haq J. (2022). Nexus between remittance outflows and economic growth in GCC countries: the mediating role of financial development. Appl. Econ. 1–13. 10.1080/00036846.2022.2139812

[B2] Aluko H. A. Aluko A. Ogunjimi F. (2022). The implications of psychological contract on employee job performance in education service delivery: a study of Ebonyi State University. Open J. Bus. Manag. 10, 978–999. 10.4236/ojbm.2022.102053

[B3] Amoah V. S. Annor F. Asumeng M. (2021). Psychological contract breach and teachers' organizational commitment: mediating roles of job embeddedness and leader-member exchange. J. Educ. Adm. 59, 634–649. 10.1108/JEA-09-2020-0201

[B4] Barnett T. Schubert E. (2002). Perceptions of the ethical work climate and covenantal relationships. J. Bus. Ethics 36, 279–290. 10.1023/A:1014042613106

[B5] Bartels A. L. Peterson S. J. Reina C. S. (2019). Understanding well-being at work: development and validation of the eudaimonic workplace well-being scale. PLoS ONE 14, e0215957. 10.1371/journal.pone.0215957 31022285 PMC6483236

[B6] Baruch Y. Rousseau D. M. (2019). Integrating psychological contracts and ecosystems in career studies and management. Acad. Manag. Ann. 13, 84–111. 10.5465/annals.2016.0103

[B7] Bathmaker S. (1999). “So, what's the deal?” the state of the psychological contract in a ‘new' university. J. Vocat. Educ. Train. 51, 265–282. 10.1080/13636829900200084

[B8] Bellou V. (2007). Shaping psychological contracts in the public and private sectors: a human resources management perspective. Int. Public Manag. J. 10, 327–349. 10.1080/1096749070151551536200579

[B9] Bless H. (2000). “The interplay of affect and cognition: the mediating role of general knowledge structures,” in Feeling and Thinking: The Role of Affect in Social Cognition, J. P. Forgas (New York, NY: Cambridge University Press), 201–222.

[B10] Bloor G. Dawson P. (1994). Understanding professional culture in organizational context. Organ. Stud. 15, 275–295. 10.1177/01708406940150020511551664

[B11] Borman W. C. Motowidlo S. M. (1993). “Expanding the criterion domain to include elements of contextual performance,” in Personnel Selection in Organizations, eds N. Schmitt, and W. C. Borman (San Francisco, CA: Wiley), 71–98.

[B12] Bunderson J. S. (2001). How work ideologies shape the psychological contracts of professional employees: doctors' responses to perceived breach. J. Organ. Behav. 22, 717–741. 10.1002/job.112

[B13] Cao J.-X. (2009). The analysis of tendency of transition from collectivism to individualism in China. Cross Cult. Commun. 5, 42–50. 10.3968/j.ccc.1923670020090504.005

[B14] Coyle-Shapiro J. A. Conway N. (2005). Exchange relationships: examining psychological contracts and perceived organizational support. J. Appl. Psychol. 90, 774–81. 10.1037/0021-9010.90.4.774 16060794

[B15] Cullinane N. Dundon T. (2006). The psychological contract: a critical review. Int. J. Manag. Rev. 8, 113–129. 10.1111/j.1468-2370.2006.00123.x

[B16] Cuyper N. Witte H. (2006). The impact of job insecurity and contract type on attitudes, well-being and behavioural reports: a psychological contract perspective. J. Occup. Organ. Psychol. 79, 395–409. 10.1348/096317905X53660

[B17] Diener E. Napa Scollon C. Lucas R. E. (2009). “The evolving concept of subjective well-being: the multifaceted nature of happiness,” in Assessing Well-Being: The Collected Works of Ed Diener, ed E. Diener (Dordrecht: Springer Netherlands), 67–100. 10.1007/978-90-481-2354-4_4

[B18] Dutton J. E. Roberts L. M. Bednar J. (2010). Pathways for positive identity construction at work: four types of positive identity and the building of social resources. Acad. Manag. Rev. 35, 265–293. 10.5465/AMR.2010.48463334

[B19] Gander M. (2022). The psychological contracts of university professional staff: expectations, obligations and benefits. Perspect.: Policy Pract. High. 27, 2–7. 10.1080/13603108.2022.2077854

[B20] Garcia-Alvarez D. Soler M. J. Achard-Braga L. (2021). Psychological well-being in teachers during and post-COVID-19: positive psychology interventions. Front. Psychol. 12, 769363. 10.3389/fpsyg.2021.769363 34975659 PMC8716601

[B21] Gerber M. Grote G. Geiser C. Raeder S. (2012). Managing psychological contracts in the era of the “new” career. Eur. J. Work. Organ. Psychol. 21, 195–221. 10.1080/1359432X.2011.55380136506968

[B22] Gottschalk L. McEachern S. (2010). The frustrated career: casual employment in higher education. Aust. Univ. Rev. 52, 37–50. Available online at: https://search.informit.org/doi/10.3316/informit.938456264757736

[B23] Han J. Yin H. Boylan M. (2016). Teacher motivation: definition, research development and implications for teachers. Cogent Educ. 3, 1217819. 10.1080/2331186X.2016.1217819

[B24] Heinz M. (2015). Why choose teaching? An international review of empirical studies exploring student teachers' career motivations and levels of commitment to teaching. Educ. Res. Eval. 21, 258–297. 10.1080/13803611.2015.1018278

[B25] Hendrawijaya A. T. Hilmi M. I. Hasan F. Imsiyah N. Indrianti D. T. (2020). Determinants of teacher performance with job satisfactions mediation. Int. J. Instr. 13, 845–860. 10.29333/iji.2020.13356a

[B26] Hui C. Lee C. Rousseau D. M. (2004). Psychological contract and organizational citizenship behavior in China: investigating generalizability and instrumentality. J. Appl. Psychol. 89, 311–21. 10.1037/0021-9010.89.2.311 15065977

[B27] Jong J. D. Schalk R. de Cuyper N. (2015). Balanced versus unbalanced psychological contracts in temporary and permanent employment: associations with employee attitudes. Manag. Organ. Rev. 5, 329–351. 10.1111/j.1740-8784.2009.00156.x

[B28] Kitwood T. (1984). Cognition and emotion in the psychology of human values. Oxford Rev. Educ. 10, 293–301. 10.1080/0305498840100306

[B29] Lee C.-H. Chen C.-W. (2021). The impact of psychological contract violation and generation difference in the workplace: an empirical study of China. Hum. Syst. Manag. 40, 825–841. 10.3233/HSM-201073

[B30] Likert R. (1932). A technique for the measurement of attitudes. Arch. Psychol. 140, 55–55.

[B31] Macneil I. R. (1985). Reflections on relational contract. Z. Gesamte Staatswiss. 141, 541–546.

[B32] Mansur K. H. M. Mucun L. Yuting Z. Yiwen C. Fu Y. (2021). The effect of psychological contract violation in employees' emotional labor strategies—mediating model with regulation. E3S Web Conf. 251, 01009. 10.1051/e3sconf/202125101009

[B33] Marks A. (2001). Developing a multiple foci conceptualization of the psychological contract. Employee Relat. 23, 454–469. 10.1108/EUM0000000005897

[B34] Motowidlo S. J. Van Scotter J. R. (1994). Evidence that task performance should be distinguished from contextual performance. J. Appl. Psychol. 79, 475–480. 10.1037/0021-9010.79.4.475 10948797

[B35] Murphy K. R. Shiarella A. H. (1997). Implications of the multidimensional nature of job performance for the validity of selection tests: multivariate frameworks for studying test validity. Pers. Psychol. 50, 823–854. 10.1111/j.1744-6570.1997.tb01484.x

[B36] O'Donohue W. Nelson L. (2009). The role of ethical values in an expanded psychological contract. J. Bus. Ethics 90, 251–263. 10.1007/s10551-009-0040-1

[B37] O'Donohue W. Sheehan C. Hecker R. Holland P. (2007). The psychological contract of knowledge workers. J. Knowl. Manag. 11, 73–82. 10.1108/13673270710738924

[B38] Pearce J. L. Rousseau D. M. (1998). Psychological contracts in organizations: understanding written and unwritten agreements. Adm. Sci. Q. 43, 184–186. 10.2307/2393595

[B39] Pöysä S. Pakarinen E. Lerkkanen M.-K. (2021). Patterns of teachers' occupational well-being during the COVID-19 pandemic: relations to experiences of exhaustion, recovery, and interactional styles of teaching. Front. Educ. 6, 699785. 10.3389/feduc.2021.699785

[B40] Pradhan R. K. Jena L. K. (2016). Employee performance at workplace: conceptual model and empirical validation. Bus. Perspect. Res. 5, 69–85. 10.1177/2278533716671630

[B41] Rousseau D. (1995). Psychological Contracts in Organizations: Understanding Written and Unwritten Agreements. Thousand Oaks, CA: SAGE. 10.4135/9781452231594

[B42] Rousseau D. M. (1989). Psychological and implied contracts in organizations. Empl. Responsib. Rights J. 2, 121–139. 10.1007/BF01384942

[B43] Rousseau D. M. (2001). Schema, promise and mutuality: the building blocks of the psychological contract. J. Occup. Organ. Psychol. 74, 511–541. 10.1348/096317901167505

[B44] Rust K. G. Mckinley W. Moon G. Edwards J. C. (2016). Ideological foundations of perceived contract breach associated with downsizing: an empirical investigation. J. Lead. Organ. Stud. 12, 37–52. 10.1177/107179190501200105

[B45] Seeck H. Parzefall M. R. (2008). Employee agency: challenges and opportunities for psychological contract theory. Personnel Rev. 37, 473–489. 10.1108/00483480810891637

[B46] Sewpersad R. Ruggunan S. Adam J. K. Krishna S. B. N. (2019). The impact of the psychological contract on academics. SAGE Open 9, 2158244019840122. 10.1177/2158244019840122

[B47] She S. Xu H. Wu Z. Tian Y. Tong Z. (2020). Dimension, content, and role of platform psychological contract: based on online ride-hailing users. Front. Psychol. 11, 2097. 10.3389/fpsyg.2020.02097 33101102 PMC7554242

[B48] Shore L. M. Barksdale K. (1998). Examining degree of balance and level of obligation in the employment relationship: a social exchange approach. J. Organ. Behav. 19, 731–744. 10.1002/(SICI)1099-1379(1998)19:1+<731::AID-JOB969>3.0.CO;2-P

[B49] Sturges J. Conway N. Guest D. Liefooghe A. (2005). Managing the career deal: the psychological contract as a framework for understanding career management, organizational commitment and work behavior. J. Organ. Behav. 26, 821–838. 10.1002/job.341

[B50] Suazo M. M. (2009). The mediating role of psychological contract violation on the relations between psychological contract breach and work-related attitudes and behaviors. J. Manag. Psychol. 24, 136–160. 10.1108/0268394091092885629975492

[B51] Tangney J. P. Stuewig J. Mashek D. J. (2007). Moral emotions and moral behavior. Annu. Rev. Psychol. 58, 345–72. 10.1146/annurev.psych.56.091103.070145 16953797 PMC3083636

[B52] Thompson J. A. Bunderson J. S. (2003). Violations of principle: ideological currency in the psychological contract. Acad. Manag. Rev. 28, 571–586. 10.2307/30040748

[B53] Thompson J. A. Hart D. W. (2006). Psychological contracts: a nano-level perspective on social contract theory. J. Bus. Ethics 68, 229–241. 10.1007/s10551-006-9012-x

[B54] Tookey M. C. (2013). The Impact of the Academic Psychological Contract on Job Performance and Satisfaction. Norwich: University of East Anglia.

[B55] Tov W. Diener E. (2009). “Culture and subjective well-being,” in Social Indicators Research Series, Vol. 38 (Dordrecht: Springer), 9–41. 10.1007/978-90-481-2352-0_2

[B56] Turnley W. (2003). The impact of psychological contract fulfillment on the performance of in-role and organizational citizenship behaviors. J. Manage. 29, 187–206. 10.1177/014920630302900204

[B57] Van Scotter J. R. Motowidlo S. J. (1996). Interpersonal facilitation and job dedication as separate facets of contextual performance. J. Appl. Psychol. 81, 525–531. 10.1037/0021-9010.81.5.525

[B58] Victor B. Cullen J. B. (1988). The organizational bases of ethical work climates. Adm. Sci. Q. 33, 101–125. 10.2307/2392857

[B59] Wright T. A. Cropanzano R. (2000). Psychological well-being and job satisfaction as predictors of job performance. J. Occup. Health Psychol. 5, 84–94. 10.1037/1076-8998.5.1.84 10658888

[B60] Wu M. Ul-Haq J. Zafar N. U. Sun H. Jiang J. (2019). Trade liberalization and informality nexus: evidence from Pakistan. J. Int. Trade Econ. Dev. 28, 732–754. 10.1080/09638199.2019.159348926743648

[B61] Ye W. Ding Y. Han X. Ye W. (2022). Pre-service teachers' teaching motivation and perceptions of teacher morality in China. Educ. Stud. 1–18. 10.1080/03055698.2022.2037406

[B62] Ye W. Zhou B. (2020). Special rank teachers' morality development in China. Prof. Dev. Educ. 49, 1–14. 10.1080/19415257.2020.1814384

[B63] You C. (2014). Analysis on the generalization of the address term “teacher” in chinese from the perspective of sociolinguistics. Theory Pract. Lang. Stud. 4, 575–580. 10.4304/tpls.4.3.575-580

[B64] Zhang L. (2022). Impact of psychological contract breach on firm's innovative performance: a moderated mediation model. Front. Psychol. 13, 970622. 10.3389/fpsyg.2022.970622 36092046 PMC9450954

[B65] Zhao H. A. O. Wayne S. J. Glibkowski B. C. Bravo J. (2007). The impact of psychological contract breach on work-related outcomes: a meta-analysis. Pers. Psychol. 60, 647–680. 10.1111/j.1744-6570.2007.00087.x

